# Supplementary figures and images for: E-cadherin endocytosis promotes non-canonical EGFR:STAT signalling to induce cell death and inhibit heterochromatinisation
Source: PLoS Genet. 2025 Jul 21;21(7):e1011781. doi: 10.1371/journal.pgen.1011781 (PMC12303393; doi:10.1371/journal.pgen.1011781)

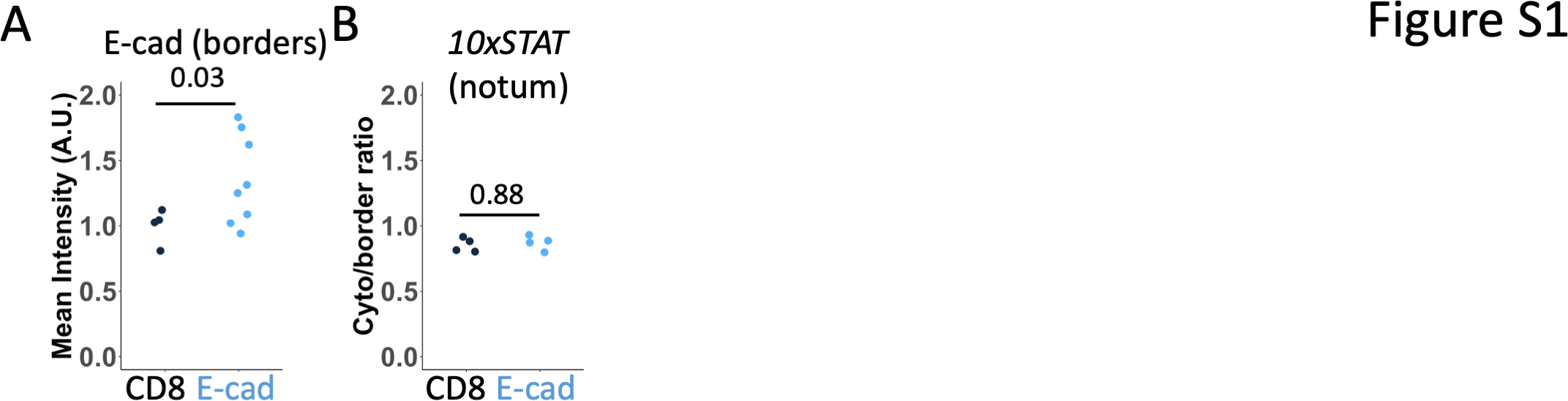

Supplement: S1 Fig — (A) Quantification of the E-cad mean intensity at cell-cell borders in wing discs expressing CD8::mCherry (left, black) and E-cad (right, blue). The Wilcoxon test was used to compare the datasets. N = 4 and 8 wing discs. The representative images and other quantifications are shown in Fig 1D–1G. (B) Quantification of 10xSTAT92E-GFP levels (10xSTAT) in the notum of wing discs expressing CD8::mCherry and E-cad::EOS. The Wilcoxon test was used to compare the datasets. N = 4 and 4 wing discs. The representative images and the quantification in the wing pouch area are shown in Fig 1C and 1H. (TIF) [file pgen.1011781.s001.tif]

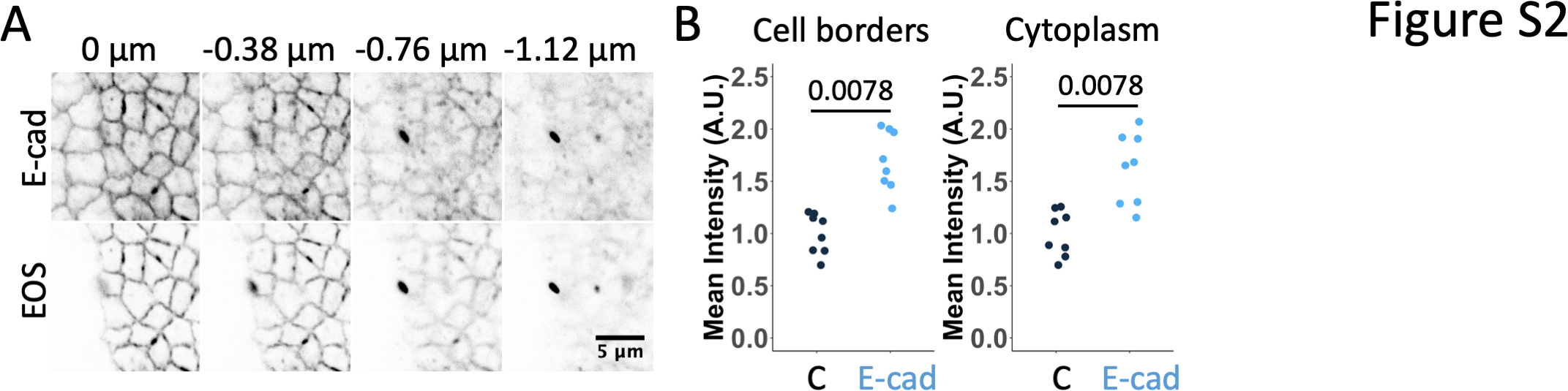

Supplement: S2 Fig — Representative images (A) and quantification (B) of cells overexpressing E-cad::EOS with ptc-Gal4 for 19 hours. Single confocal z-sections of the E-cad antibody staining (A, top row) and native EOS fluorescence (A, bottom row) are depicted with the depth at the middle of the adherens junctions set to 0 and decreasing as progressing from the apical to basal direction. The paired-samples Wilcoxon test was used to compare expressing (E-cad, blue in B) and non-expressing areas in same wing discs (C, black in B). N = 8 wing discs. (TIF) [file pgen.1011781.s002.tif]

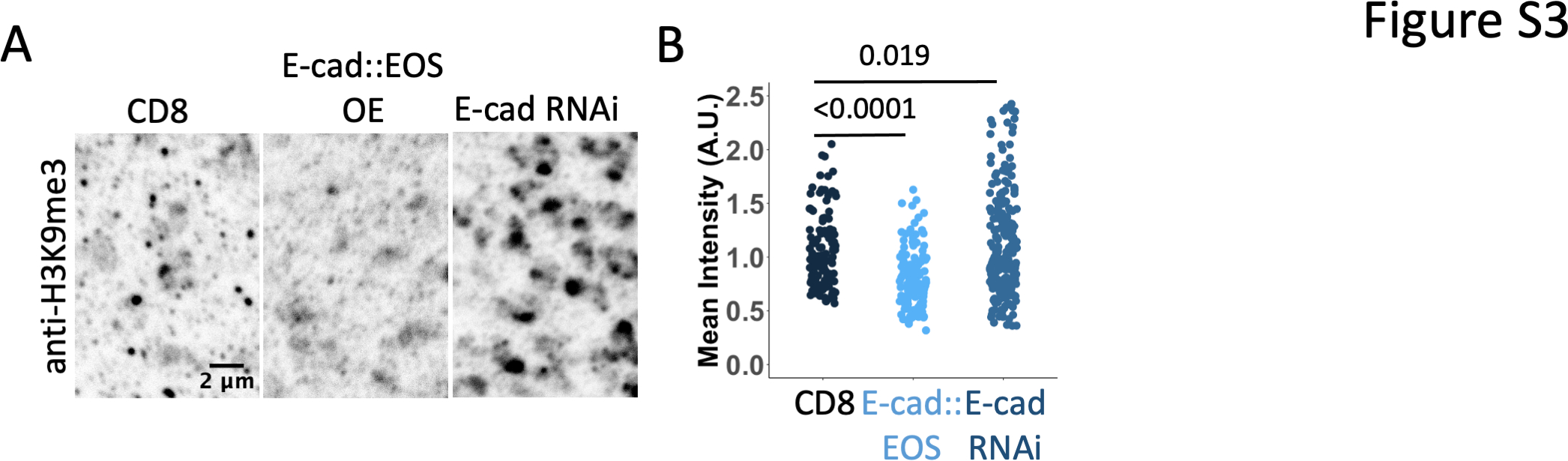

Supplement: S3 Fig — Representative images (A) and quantification (B) of H3K9me3 (antibody staining) in wing discs with E-cad::EOS overexpression (OE, middle), E-cad knockdown (RNAi) or overexpression of CD8::mCherry as control (left) for 24 hours using ptc-Gal4. Each dot represents a single puncta. One-way ANOVA and post-hoc t-test with false discovery rate p-value correction were used in B. N = 136/3, 125/3 and 228/6 (punctum/wing disc). Similar data for HP1 is in Fig 2B and 2C. (TIF) [file pgen.1011781.s003.tif]

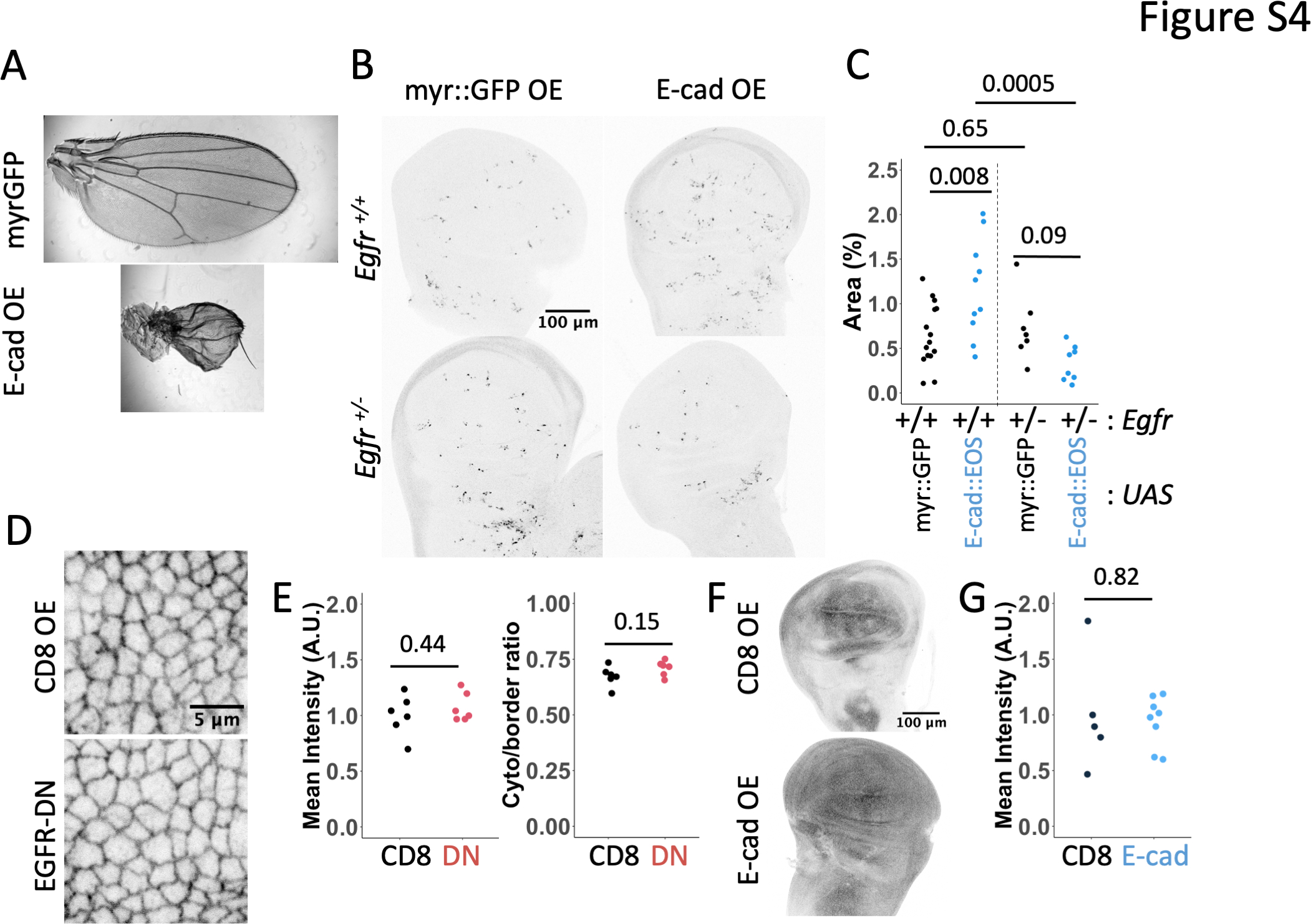

Supplement: S4 Fig — (A) Representative images of adult wings expressing two copies of myr::GFP (top) and untagged E-cad (bottom). (B-C) Representative images (B) and quantification (C) of Dcp-1 staining in wing discs expressing myr::GFP (B, left) or untagged full-length E-cad (B, right column) in the presence of two functional copies (B, top row) or one copy (B, bottom row) of the Egfr gene. The MS1096-Gal4 driver was used. Two-way ANOVA and post-hoc t-test with false discovery rate p-value correction were used. N = 15, 7, 10 and 7, left-to-right. p = 0.003 for the interaction between untagged E-cad overexpression and Egfr copy number. Similar datasets for E-cad::EOS are in Fig 3A–3D. (D-E) Representative images (D) and quantification (E) of mean cell-cell border levels (E, left) and the ratio between the cytoplasm and cell-cell border levels (E, right) of E-cad::GFP expressed from a ubiquitous promoter in control wing discs (D, left, E, black) and those expressing EGFR-DN (D, right, E, red). The Wilcoxon test was used to compare the datasets. N = 6 and 6 wing discs. Similar dataset for Egfr heterozygotes is in Fig 3E and 3F. (F-G) Representative images (F) and quantification (G) of levels of phosphorylated ERK (pERK) visualised with antibody staining in control wing discs (F, left, G, black) and those overexpressing E-cad::EOS (F, right, G, blue). The Wilcoxon test was used to compare the datasets. N = 5 and 8 wing discs. (TIF) [file pgen.1011781.s004.tif]

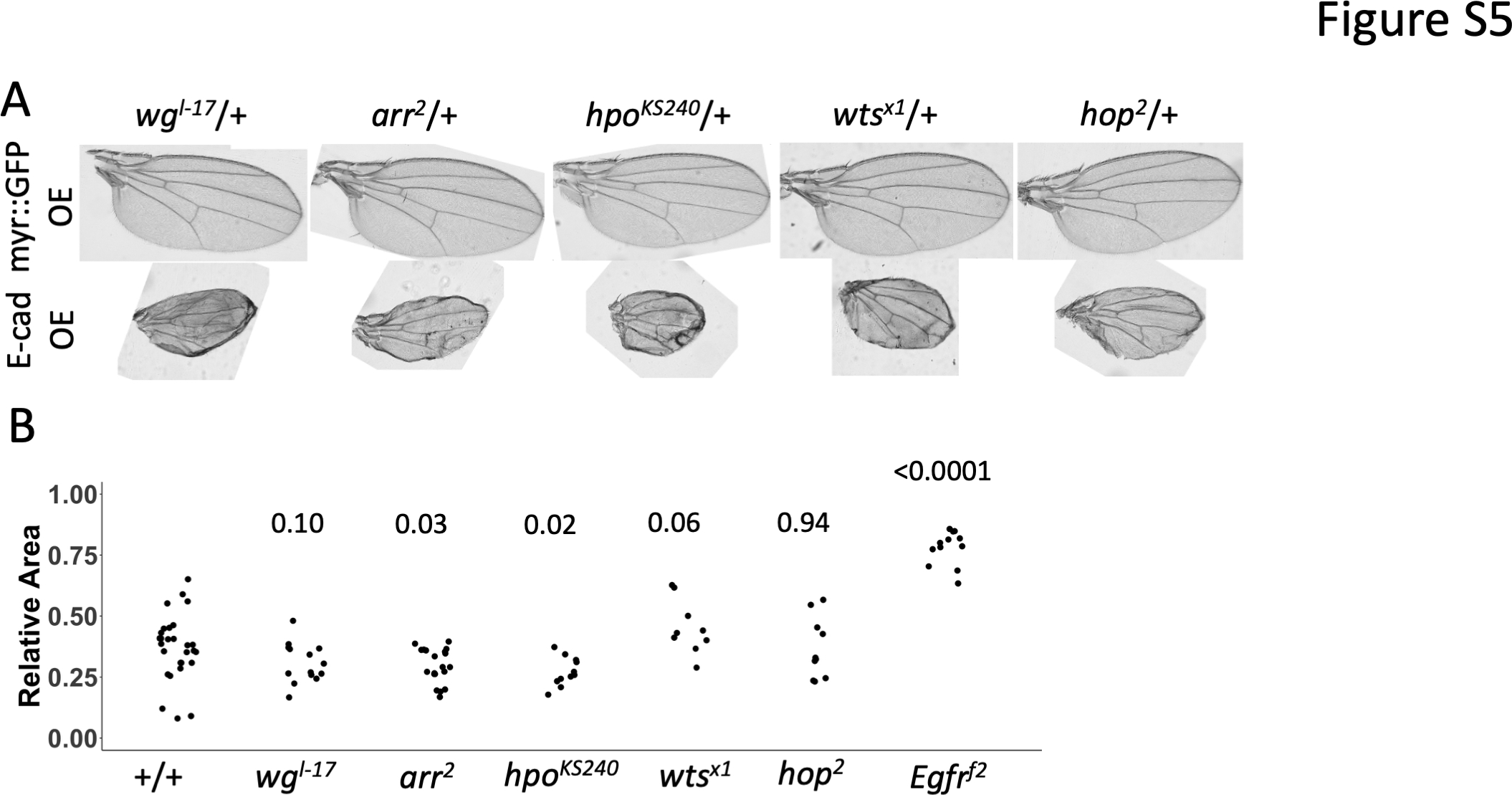

Supplement: S5 Fig — Representative images (A) and area quantification of adult wings (B) expressing either myr::GFP (A, top row) or E-cad::EOS (A, bottom row) in the presence of two copies (A, top) or one functional copy (A, bottom) of the following genes: wingless (wgl-17 allele), arrow (arr2 allele, encoding a co-receptor of wg), hippo (hpoKS240 allele), warts (wtsx1 allele), and hopscotch (hop2 allele, encoding for the single Drosophila JAK protein). The quantification is shown as a relative area to corresponding controls (one gene copy without E-cad::EOS overexpression). One-way ANOVA and post-hoc t-test with false discovery rate p-value correction were used. N = 14/12, 21/12, 11/14, 9/4 and 10/12 (one copy/two copies), left-to-right. (TIF) [file pgen.1011781.s005.tif]
