# Supplementary material for: E-cadherin endocytosis promotes non-canonical EGFR:STAT signalling to induce cell death and inhibit heterochromatinisation
Source: PLoS Genet. 2025 Jul 21;21(7):e1011781. doi: 10.1371/journal.pgen.1011781 (PMC12303393; doi:10.1371/journal.pgen.1011781)
Supplement: Attachment — Submitted filename: PGENETICS-D-24-01504 reviewers response.docx [file pgen.1011781.s008.docx]

**PGENETICS-D-24-01504** Remirez-Moreno *et al.* E-cadherin endocytosis promotes non-canonical EGFR:STAT signalling to induce cell death and inhibit heterochromatinisation

**Response to reviewers’ comments.**

Reviewers’ comments are in *italics*, and our response is in Roman.

We would like to thank all three reviewers for their positive comments that highlighted the significance and impact of our findings. For example, reviewer #2 points out that “*This is a very interesting concept, and the paper is written clearly. These molecules and signaling pathways are of great interest to many, so the results could be quite impactful*” and reviewer #1 comments that “*the manuscript presents a well-executed study with significant implications for understanding the biological functions and regulations of non-canonical STAT signaling pathways.*”

We are pleased to submit a revised version of the manuscript that includes new evidence for our model based on the experiments suggested by reviewers. We have also made several minor adjustments to the original text to enhance clarity and correct grammatical errors. Below, we address the individual concerns raised, starting with the major concerns brought up by Reviewer #2, as this reviewer raised the most points.

**Major concerns. Reviewer #2.**

This reviewer states that “*Generally, I have concerns about making conclusions about direct pathway interactions based on the genetic manipulations carried out in this paper, which are likely to have widespread effects.”* While the reviewer makes a good point, the fact remains that genetic manipulations analysed in an in vivo system can be a uniquely powerful and predictive experimental approach. For example, much of the EGFR/Ras/Raf/ERK pathway was identified and functionally characterised in *Drosophila* in the 1990s using exactly this approach (see, for example, the review PMID: [12485984](https://pubmed.ncbi.nlm.nih.gov/12485984/)). Furthermore, the insights into complex relationships in an in vivo context gained using this approach have proved to be accurate, robust and largely correct. However, there are certainly caveats in any approach, and to make it clear that this is a developmental genetics study rather than a direct biochemical demonstration of interactions, we have now added a clarification on page 4, lines 22-24.

The reviewer adds, “*So some of the downstream genes may be regulated indirectly, not just by these pathways as the paper implies.*” We agree and do not intend to suggest that our pathway of interest directly regulates any specific individual change in gene expression. In the revised version of the manuscript, we took additional care to avoid implying the direct regulation, for instance by replacing “transcriptional targets” with “patterns of gene expression changes” on page 8, lines 22-23. Nevertheless, we posit that the overall similarity in the ‘fingerprint’ or pattern of gene expression change provides compelling evidence to suggest that STAT and E-cad work together in an opposing direction to HP1. As such, additional RNA-seq timepoints would not necessarily help resolve this question, but rather, it would require approaches such as ChIP-seq, which are outside the scope of this study.

The reviewer questions the existence of the complex between E-cad, STAT and EGFR and suggests “*these proteins are generally found at the plasma membrane, but aside from that, we cannot tell if they are close enough to interact. Other methods might resolve this and would provide a more convincing argument, such as proximity ligation assays, which they seem to have most of the tools to do.*”

Unfortunately, the proximity ligation assay is not an option in this case. E-cad antibody recognises its extracellular domain, so it is unlikely to be close enough to the intracellular STAT92E, while no antibody recognising STAT92E is publicly available. Furthermore, our working hypothesis is that STAT92E is likely to bind to E-cad via the large scaffold protein Par-3. However, we have made three attempts to immunoprecipitate E-cad with EGFR::GFP and STAT92E::GFP from wing imaginal discs over the recent months - a sequence of ultimately unsuccessful experiments inspired directly by this comment. We suspect this failure is a result of technical limitations, as we could not even detect E-cad in the precipitate, likely due to the extremely low amount of protein that can be recovered from dissected wing imaginal discs. Despite this, we feel that the balance of evidence continues to suggest that the three proteins are likely to form a complex based on four pieces of evidence: (a) we see them co-localising in vivo (Fig 1D-F and Fig. 3A-C), (b) E-cad and EGFR have previously been shown to co-immunoprecipitate in *Drosophila* (PMID: [12163402](https://pubmed.ncbi.nlm.nih.gov/12485984/), [15057284](https://pubmed.ncbi.nlm.nih.gov/15057284/)), (c) STAT92E coimmunoprecipitates with Par3, and Par3 – with E-cad in *Drosophila* (PMID: [23751496](https://pubmed.ncbi.nlm.nih.gov/23751496/), [23462467](https://pubmed.ncbi.nlm.nih.gov/23462467/)), and (d) the highly conserved human homologues CDH1 (E-cad) and STAT1 were found in close proximity in human cells as shown using proximity biotinylation and quantitative proteomics (PMID: [25468996](https://pubmed.ncbi.nlm.nih.gov/25468996/)). We have added a reference to point (d) in support of complex conservation at the end of the discussion section (page 10, lines 24-49).

*The authors argue that loss at the membrane is a result of endocytosis, but this is never shown directly… the use of mutants that block endocytosis to reverse their appearance (or directly showing images of the result attributed to the literature that p120-catenin would block this).*

Both other groups and our data have previously demonstrated that E-cad endocytosis is blocked by p120-catenin overexpression (PMID: [32665321](https://pubmed.ncbi.nlm.nih.gov/32665321/), [14610056](https://pubmed.ncbi.nlm.nih.gov/14610056/)), because p120-catenin binds to the E-cad intracellular domain and prevents E-cad from being endocytosed (PMID: [23071156](https://pubmed.ncbi.nlm.nih.gov/23071156/)). In the revised manuscript on Fig. 3E-G and page 6, lines 29-35, we have added new data to show that p120-catenin co-overexpression with E-cad prevents the relocalisation of EGFR::GFP observed upon E-cad overexpression alone. This result demonstrates that E-cad endocytosis is causative for the relocalisation of EGFR by E-cad overexpression in Fig. 3A.

*The authors show that HP1 levels in the nucleus changes in response to lower E-cadherin, as does the amount of H3K9me3. However, HP1 can work with many partners, so this does not necessarily mean STAT is involved. Perhaps they can look for co-localization with STAT and HP1 at a high resolution, or evaluate the genetic effects of HP1 in their assays combined with genetic changes in STAT levels.*

This is a fair point, and we certainly do not intend to suggest that STAT is the only regulator of HP1 activity in vivo. While excluding the unknown is always a challenge, we have sought to address this question by adding a new experiment in which we demonstrate that E-cad overexpression reduces the nuclear/cytoplasmic ratio of STAT::GFP levels (Fig. 2D-F, page 6, lines 15-19). Therefore, reduced nuclear HP1 levels correlate with reduced nuclear/cytoplasmic STAT ratio following E-cad overexpression. Considering the well-established role of STAT in stabilising HP1 on heterochromatin (PMID: [18344984](https://pubmed.ncbi.nlm.nih.gov/18344984/)), this additional data further supports our model whereby E-cad overexpression outcompetes STAT from the nucleus and HP1 binding. An expanded section discussing the evidence supporting these relationships has also been added to the discussion section to clarify this point further (page 9, line 34-48).

*The data with STAT suppressing this phenotype are a bit weak since loss of stat on its own resulted in a larger wing, so this may be separable.*

The reviewer is correct that reducing the *stat92E* gene dose alone leads to a larger wing. For this reason, we performed a 2-way ANOVA analysis, as it allows investigating both the individual effects of each factor and the interaction between them. The p = 0.02 for the interaction between two factors – E-cad::EOS overexpression and *stat92E* copy number – is shown in the figure legend (Fig. 6A-B) and suggests that the increase in wing size cannot be explained by the *STAT* gene dose alone.

*Loss of HP1 also induced a small wing, but this was not tested in combination with E-cad or STAT or evaluated for effects on cell death.*

We thank the reviewer for pointing this out and have undertaken new experiments to test for these potential interactions. New data describing the genetic interaction between HP1 and E-cad has been added (see new Fig. 6E-F, page 7, lines 31-33) and shows that the simultaneous overexpression of the untagged full-length E-cad and HP1 RNAi combined to reduce the adult wing size synergistically.

*For POSH heterozygotes and E-cad overexpression, only size was measured, but the number of apoptotic cells would need to be counted here as well to make the argument that this pathway is mediating the effect through cell death.*

This is indeed an important experiment given our focus on apoptosis. As such, we have undertaken this experiment as suggested by this reviewer. Reassuringly, this new data demonstrates that reducing the *POSH* copy number prevents the increase in the amount of apoptosis caused by E-cad overexpression (see Fig. 8C-D, page 8, lines 13-14). This result, alongside our new data on *basket* and p35 (see below), supports that the pathway mediates its effect through apoptosis.

*Overexpression with the STAT Y740F mutant and STAT wt might have differential effects on these phenotypes*

We thank the reviewer for this suggestion, which seeks to dissect the different potential canonical and non-canonical activities predicted for these two STAT92E transgenes. Unfortunately, such an experiment would be unlikely to provide a definitive insight. This is because the overexpression of STAT92E(wt) would have the same non-canonical functions as STAT92E[Y704F], but the overexpression of STAT92E(wt) would additionally activate the canonical signalling (PMID: [32917740](https://pubmed.ncbi.nlm.nih.gov/32917740/)). This activation will produce additional phenotypic changes that may interact with non-canonical functions in an unpredictable manner. As a result, the subsequent interpretation of such an experiment would be complex, requiring further validation and beyond the scope of this paper’s revisions. For that reason, we decided not to carry out this experiment. However, our new data on the interaction between E-cad and HP1-RNAi (Fig. 6E-F, page 7, lines 31-33) and the effects of E-cad on STAT nuclear/cytoplasmic levels (Fig. 2D-F, page 6, lines 15-19) support our conclusion about the “*competition for STAT in non-canonical pathways downstream of E-cadherin*”.

*To substantiate the argument that the wing effects of high E-cad are mediated by JNK signaling (and to help validate the idea that the RNAseq data is informative), the authors should look at JNK activation directly, either by assaying changes in expression of these genes/proteins or evaluating an available reporter (one option may be puc-LacZ).*

We thank the reviewer for this suggestion, seeking to examine the mechanistic basis of the apoptotic regulation we describe in this paper, which the reviewer agrees may be mediated by the JNK pathway. Consistent with this hypothesis, we find that the expression of the JNK pathway target genes – *puckered* and *cheerio* (<https://www.sdbonline.org/sites/fly/aignfam/jnkpath.htm>) – is increased following overexpression of STAT92E[Y704F] in our RNA-seq dataset (adjusted p = 1.6E-05 and p = 0.01). We have attempted to demonstrate this effect in vivo using reporters as suggested by the reviewer. Unfortunately, the fly line with double-balanced *MS1096*-Gal4 with *puc*-LacZ has very poor viability, and we were therefore unable to carry out the experiment proposed by the reviewer. However, we have added new findings on the genetic interaction between E-cad and *bsk* (encoding for the only *Drosophila* JNK, Fig. 7C-D, page 8, lines 5-9*)* and the rescue of the small wing phenotype by blocking apoptosis using p35 (Fig. 4E-F, page 6, lines 43-47). Taken together, we feel that these transcriptional data, together with the genetic interactions, substantiate our claims regarding the effects being mediated by JNK signalling and cell death.

**Minor points**:

Reviewer #1:

*Figure 1. Should provide an estimate of the levels of changes in HP1 and STAT after transgene overexpression.*

Log_2_ values for fold changes in HP1 and STAT are -100 and 27, indicating strong knockdown and overexpression on mRNA levels. We have added the fold change upon our genetic manipulations and relevant adjusted p-values on page 4, lines 45-46, and page 5, line 45.

*Discussion: Add more discussion on the potential broader impact of the findings e.g., in tumor suppression and potential therapeutic applications.*

In light of the new results added to the manuscript, we have expanded the discussion on the potential for our findings to represent a novel tumour suppressor activity associated with epithelial-to-mesenchymal transitions, given the evolutionary conservation of the pathways involved (page 10, lines 9-14 and lines 24-29).

*I suggest the authors not claim to see localization to adherens Junctions (AJ), and relabel graphs to refer to cell membrane localization*

We replaced “AJ” with “borders” on all graphs as suggested.

*The correlation analyses are somewhat hard to understand (MCC) even after reading the methods. What is the x-axis? Is this in different genetic conditions – it appears in the panel 1D that STAT is just about everywhere, so it is a correlation if 24% of this is at the membrane (1E)? In Fig panel 2F, why is this a lower coefficient (3-7%) than in the prior figure when it appears in the figure to be more often co-localized?*

We thank the reviewer for pointing out this inconsistency. After investigating it further, we found that the Coloc 2 plug-in in Fiji reports MCC (overall % of overlapping pixels from all pixels above the threshold) rather than MC_diff_ (% of overlapping pixels normalised to the expected % from random localisation). We have now manually recalculated MCC_diff_ for STAT::GFP as described in the new materials and methods section (Fig. 1E). Importantly, in Fig. 3C, the co-localisation was only measured in the cytoplasm. We clarified this on the Y-axis, added descriptions of the X-axes to both graphs, and elaborated on the description of how MCC_diff_ was measured in the Materials and Methods section.

*Figure panels 1A and 2A seem redundant – both are probably not needed.*

We agree with the reviewer and have replaced Fig. 2A with the correlation in gene expression changes upon E-cad and STAT[Y704F] overexpression that highlights the components of the JNK pathway.

**Reviewer #3:**

1) *Since it is important to compare fly lines containing the same number of UAS lines between control vs experimental genotype (as expression levels change depending on the number of UAS lines a fly has), authors could consider providing UAS expressing controls for the above mentioned examples as well.*

We agree with the reviewer, and the Gal4/UAS dosage was controlled in all our experiments. We have now provided information about control UAS lines for all figure panels.

2) *It would be interesting to see the effect on imaginal discs and wing size upon overexpression of a EGFR constitutively active construct in combination with E-cad::EOS and without, as this will provide a different type of arguments on the E-cad/EGFR interaction. If the effect of EGFR-CA is too strong, authors can overexpress a wild type EGFR construct.*

This is a good suggestion, and we have undertaken several experiments using these constructs. Unfortunately, the effect of EGFR-CA is indeed too strong to draw any conclusions. In contrast, the overexpression of EGFR-wt alone reduced the adult wing size in addition to expected phenotypes, such as wing blistering and vein defects. EGFR-wt co-overexpression with the untagged full-length E-cad produces the same adult wing size as EGFR-wt overexpression alone (Fig. R1A-B). It is impossible to interpret the result of this experiment, as we do not know the mechanism of EGFR-wt wing size reduction. At the same time, we found that E-cad co-overexpression rescued some of the EGFR-wt induced phenotypes (Fig. R1C-D), suggesting that E-cad antagonises the canonical EGF-mediated EGFR signalling. This possibility is not unlikely as E-cad and EGF bind the same domain of EGFR (PMID: [15057284](https://pubmed.ncbi.nlm.nih.gov/15057284/)). While these findings are outside this manuscript's scope, they support another ongoing project and will be the subject of a future publication.

**Figure R1. The interaction between E-cad and EGFR(wt) overexpression.** (A-B) Representative images and quantification of the adult wing sizes. N = you 34, 26, 39, 36 left-to-right. Two-way ANOVA and post-hoc t-test with false discovery rate p-value correction were used. (C-D) The % of wings containing blisters (C, N = 39 and 36) and the % of L5 vein length being widened (D, N = 21 and 24) upon EGFR-wt co-overexpression with either myr::GFP (left) or untagged full-length E-cad (right). The Chi-square (C) and Wilcoxon (D) tests were used.

3) *Authors should attempt to strengthen their arguments here by testing the classical Drosophila JNK basket (via RNAi or dominant negative constructs). If experimentally easy, authors can also compare the levels of puckered (via established TRE or puc-GFP lines) before and after rescue in wing imaginal discs.*

We thank the reviewer for this suggestion to enhance our story, and we have added the data on the rescue of the E-cad::EOS overexpression adult wing size phenotype by reducing *basket* gene copy number (Fig. 7C-D), which supports the contribution of JNK activation in the small wing phenotype. As explained above in the response to Reviewer 2, unfortunately, it was not possible to further strengthen the argument with *puc*-LacZ due to the low viability of the double-balanced *MS1096*-Gal4;;*puc*-LacZ line.

4) *The authors link the activation of apoptosis upon E-cad overexpression (through the non-canonical EGFR::STAT pathway) to a tumour-suppressive, cancer-preventing mechanism… Authors can modify the text to clearly separate experimental evidence from hypothesis-driven conclusions or provide experimental evidence to support this e.g. by knocking down or removing gene copies of pro-apoptotic or apoptotic genes and see whether this leads to tumour formation, overgrowths or benign over-proliferation that disrupts wing development.*

We thank the reviewer for this suggestion. While inhibiting apoptosis upon E-cad::EOS overexpression did not lead to tumour formation and larval wing discs appeared normal (not shown), this manipulation rescued the small wing phenotype, enabling us to confirm the direct link between apoptosis and this phenotype (Fig. 4E-F). We have clarified where we are discussing hypotheses and where we are referring to experimental data (e.g., the last sentence in the abstract), and added a discussion on page 10 about the hypothesised role of this mechanism.

5) *Figures 4, 5, and Supplementary Figures are missing descriptive titles that would facilitate the readers.*

We added descriptive titles for all figures.

6) *Figure 2F is missing an axis description, in Figures 3H and S4E please add the genotypes as well, as it helps readers grasp the experiment quicker.*

We added the axis description to Fig. 2F (now Fig. 3C) and genotypes to Fig. 3H and S4E (now Fig. 5F and S4E).
